# *“Candidatus* Trichorickettsia mobilis”, a* Rickettsiales* bacterium, can be transiently transferred from the unicellular eukaryote* Paramecium* to the planarian* Dugesia japonica*

**DOI:** 10.7717/peerj.8977

**Published:** 2020-04-23

**Authors:** Letizia Modeo, Alessandra Salvetti, Leonardo Rossi, Michele Castelli, Franziska Szokoli, Sascha Krenek, Valentina Serra, Elena Sabaneyeva, Graziano Di Giuseppe, Sergei I. Fokin, Franco Verni, Giulio Petroni

**Affiliations:** 1 Department of Biology, University of Pisa, Pisa, Italy; 2 CIME, Centro Interdipartimentale di Microscopia Elettronica, University of Pisa, Pisa, Italy; 3 CISUP, Centro per l’Integrazione della Strumentazione, University of Pisa, Pisa, Italy; 4 Department of Clinical and Experimental Medicine, University of Pisa, Pisa, Italy; 5 Centro Romeo ed Enrica Invernizzi Ricerca Pediatrica, Department of Biosciences, University of Milan, Milan, Italy; 6 Institute of Hydrobiology, Dresden University of Technology, Dresden, Germany; 7 Department of River Ecology, Helmholtz Center for Environmental Research—UFZ, Magdeburg, Germany; 8 Department of Cytology and Histology, Faculty of Biology, Saint Petersburg State University, Saint Petersburg, Russia; 9 Department of Invertebrate Zoology, Saint Petersburg State University, Saint Petersburg, Russia

**Keywords:** Planarians, Molecular characterization, Rickettsiaceae, *Paramecium multimicronucleatum*, “*Candidatus* Trichorickettsia”, FISH, Vector-borne diseases, *Rickettsia*-like organism (RLO) endosymbiont, Ultrastructure, Transfection experiments

## Abstract

Most of the microorganisms responsible for vector-borne diseases (VBD) have hematophagous arthropods as vector/reservoir. Recently, many new species of microorganisms phylogenetically related to agents of VBD were found in a variety of aquatic eukaryotic hosts; in particular, numerous new bacterial species related to the genus *Rickettsia* (*Alphaproteobacteria*, *Rickettsiales*) were discovered in protist ciliates and other unicellular eukaryotes. Although their pathogenicity for humans and terrestrial animals is not known, several indirect indications exist that these bacteria might act as etiological agents of possible VBD of aquatic organisms, with protists as vectors. In the present study, a novel strain of the *Rickettsia*-Like Organism (RLO) endosymbiont “*Candidatus* (*Ca.*) Trichorickettsia mobilis” was identified in the macronucleus of the ciliate *Paramecium multimicronucleatum*. We performed transfection experiments of this RLO to planarians (*Dugesia japonica*) per *os*. Indeed, the latter is a widely used model system for studying bacteria pathogenic to humans and other Metazoa. In transfection experiments, homogenized paramecia were added to food of antibiotic-treated planarians. Treated and non-treated (i.e. control) planarians were investigated at day 1, 3, and 7 after feeding for endosymbiont presence by means of PCR and ultrastructural analyses. Obtained results were fully concordant and suggest that this RLO endosymbiont can be transiently transferred from ciliates to metazoans, being detected up to day 7 in treated planarians’ enterocytes. Our findings might offer insights into the potential role of ciliates or other protists as putative vectors for diseases caused by *Rickettsiales* or other RLOs and occurring in fish farms or in the wild.

## Introduction

Bacteria of order *Rickettsiales* (*Alphaproteobacteria*) live in an obligate association with a wide range of eukaryotes (Dumler et al., 2001; Taylor, Bandi & Hoerauf, 2005; Perlman, Hunter & Zchori-Fein, 2006; Weinert et al., 2009; Gillespie et al., 2012; Dumler & Walker, 2015; Castelli, Sassera & Petroni, 2016; Chan et al., 2018), and, for their vast majority, are localized intracellularly, although the case of an extracellular *Rickettsiales* bacterium was recently documented (Castelli et al., 2019a). *Rickettsiales* are widely studied for their involvement in medical and veterinary fields. Indeed, many of them (e.g., *Rickettsia* spp., *Anaplasma* spp., *Ehrlichia* spp., and *Orientia tsutsugamushi*) are vectored by lice, ticks and mites, and cause mild to severe disease (Dumler et al., 2001; Dumler & Walker, 2015), such as epidemic typhus, Rocky Mountain spotted fever (Raoult & Roux, 1997; Walker & Ismail, 2008; McQuiston & Paddock, 2012), anaplasmosis, ehrlichiosis (Dumler et al., 2007; Rikihisa, 2010), heartwater (Allsopp, 2015), and scrub typhus (Ge & Rikihisa, 2011).

In the last two decades, many new genera and species of *Rickettsiales* were found as symbionts (with “symbiosis” simply meaning the even-temporary association of organisms from different species) in a variety of other non-vector eukaryotic hosts, both from terrestrial and aquatic environments (reviewed in Perlman, Hunter & Zchori-Fein, 2006; Weinert et al., 2009; Gillespie et al., 2012; Castelli, Sassera & Petroni, 2016). Numerous such novel bacterial species were retrieved in aquatic protists (Kawafune et al., 2015; Schulz et al., 2016; Yang, Narechania & Kim, 2016; Hess, 2017; Yurchenko et al., 2018), including, notably, parasitic (Sun et al., 2009; Zaila et al., 2017) and various free-living ciliates (Ferrantini et al., 2009; Boscaro et al., 2013; Schrallhammer et al., 2013; Vannini et al., 2014; Senra et al., 2016; Szokoli et al., 2016a; Szokoli et al., 2016b; Castelli et al., 2019b; Lanzoni et al., 2019).

Such findings strongly suggest the ability of those “aquatic” *Rickettsiales* to perform horizontal transmission including host species shift, although mostly from indirect evidence, i.e., closely related bacteria in hosts as different as ciliates, hydra (Fraune & Bosch, 2007), corals (Sunagawa et al., 2009), ring worms (Murakami et al., 2017), ascidians (Kwan & Schmidt, 2013; Dishaw et al., 2014), placozoans (Driscoll et al., 2013), as well as incongruent host and symbionts phylogenies (Perlman, Hunter & Zchori-Fein, 2006; Epis et al., 2008; Driscoll et al., 2013). In few cases, experimental interspecific transfer between unicellular hosts, although no stable infection was documented (Schulz et al., 2016; Senra et al., 2016).

In most cases the relationships between *Rickettsiales* associated with aquatic eukaryotes and their hosts were not clarified in detail (Castelli, Sassera & Petroni, 2016), with the exception of “*Candidatus* (*Ca*.) Xenohaliotis californiensis”, which is considered the cause of the withering syndrome in its abalone hosts (Friedman et al., 2000; Crosson et al., 2014). It has been hypothesized by different authors that bacteria harbored by aquatic protists might constitute etiological agents of possible Vector Borne Diseases (VBD) of aquatic animals (Barker & Brown, 1994; Taylor-Mulneix et al., 2017). These include the numerous cases of epidemics caused by *Rickettsia*-Like Organisms (RLOs; i.e., intracellular bacteria morphologically similar to *Rickettsia*) determining an increasing number of massive deaths in intensive aquaculture facilities during the last years (Gollas-Galván et al., 2014), including mollusks (Friedman et al., 2000; Sun & Wu, 2004; Ross et al., 2018), crustaceans (Loy et al., 1996; Longshaw, 2011; Wang, 2011) and fishes (Athanassopoulou et al., 2004; Corbeil, Hyatt & Crane, 2005; Timur et al., 2013). Although many RLOs were actually shown to be phylogenetically unrelated to *Rickettsiales* (e.g., *Gammaproteobacteria*) (Fryer et al., 1992; Tan & Owens, 2000; Longshaw, 2011), at least in one case available data indicate a probable connection between a truly *Rickettsiales* bacterium and fish disease. In detail, a “*Ca*. Midichloria mitochondrii”-related bacterium was linked to the red-mark syndrome in rainbow trout (Lloyd et al., 2008; Cafiso et al., 2015). Most importantly, although a transmission route was not directly proven, the same bacterium was found in association with the ciliate *Ichthyophthirius multifiliis* (Zaila et al., 2017), which is indeed a fish parasite. Interestingly, even other *Rickettsiales* symbionts (“*Ca*. Megaira” genus) can be found in the same ciliate host species, which might suggest a potential transmission route for these symbionts as well (Sun et al., 2009; Zaila et al., 2017).

Taking into account such premises, in order to investigate the hypothesis that protists can act as natural reservoir for potentially pathogenic bacteria (Barker & Brown, 1994; Gao, Harb & Abu Kwaik, 1997; Görtz & Brigge, 1998; Harb, Gao & Abu Kwaik, 2000; Horn & Wagner, 2004; Molmeret et al., 2005; Ferrantini et al., 2009; Taylor-Mulneix et al., 2017), we experimentally tested the possibility that endosymbionts of ciliates could be per *os* transferred to aquatic Metazoa, such as planarians. The *Rickettsiales* “*Ca.* Trichorickettsia” was chosen as candidate for transfection experiments as it shows a broad ciliate host range, infecting different cell compartment (i.e., cytoplasm and nucleus) of multiple ciliates (Vannini et al., 2014; Sabaneyeva et al., 2018). Additionally, in many hosts, it is covered by long flagella and is able to actively move (Vannini et al., 2014; Sabaneyeva et al., 2018). Thus, for our research purpose, as donor host in transfection experiments, we employed the *P. multimicronucleatum* strain US_Bl 16I1, which was herein newly characterized and shown to bear a flagellated “*Ca*. Trichorickettsia mobilis” symbiont. On the other side, we selected the freshwater planarian *Dugesia japonica* because it is a benthic organism, living in mud and under rocks in ponds and streams. Planarians are zoophages or carnivorous animals, but ingest also detritus, fungi, and bacteria (González-Estévez, 2009). Thus, they may encounter in their environment a large variety of microbes (Petersen, 2014), including ciliates possibly hosting endosymbionts. Planarians have always been considered an important model for studying stem cells and regeneration (Rossi et al., 2008), but recently they became also important for studying the natural immunity system of Metazoa (Abnave et al., 2014; Conti, Abnave & Ghigo, 2014; Torre & Ghigo, 2015; Arnold et al., 2016; Torre et al., 2017; Tsoumtsa et al., 2017). Based on all these considerations, they appeared a suitable model for such experiments.

Bacterial endosymbiont transfer from *P. multimicronucleatum* to planarians was investigated by checking for the presence of “*Ca*. Trichorickettsia mobilis” in tissues of ciliate-fed planarians by means of PCR and Transmission Electron Microscopy (TEM) at day 1, 3, and 7 after feeding. The collected data are indicative of the ability of the endosymbiont to temporarily survive and divide in the planarian intestine, and to possibly escape from planarian phagosomes.

## Materials & Methods

### Ciliate host isolation, culturing, and identification

*Paramecium multimicronucleatum* monoclonal strain US_Bl 16I1 was established from a cell isolated from a freshwater sample collected from the Yellowwood Lake (39°11′29,0″N, 86°20′31,4″W), IN, USA and cultivated in San Benedetto mineral water (San Benedetto S. p. A., Italy).

The strain was maintained in the laboratory in an incubator at a temperature of 19 ± 1 °C and on a 12:12 h light/dark cycle (light source: NATURAL L36W/76 and FLORA L36W/77 neon tubes, OSRAM, Berlin, Germany). Instead of using bacteria as common food source for paramecia (Krenek, Berendonk & Petzoldt, 2011), cells were fed monoclonal cultures of flagellated green algae, i.e., *Chlorogonium* sp. (freshwater) or, alternatively, *Dunaliella tertiolecta* (brackish water, 1‰ of salinity) to minimize bacterial load in cell cultures. They were fed two to three times per week to obtain a mass culture (1.5 L) (Castelli et al., 2015) suitable for transfection experiments. Living ciliates were observed with a Leitz (Weitzlar, Germany) microscope equipped with Differential Interference Contrast (DIC) at a magnification of 100–1250× for general morphology and swimming behavior according to Modeo et al. (2013a) and Nitla et al., (2019). DIC observation of ciliates was also aimed at checking for macronuclear endosymbiont motility. Feulgen-stained ciliates were observed to obtain information on the nuclear apparatus. Microscope images were captured with a digital camera (Canon PowerShot S45). Cell measurements were performed using ImageJ (https://imagej.nih.gov/ij/). Morphological identification was conducted according to literature data (Fokin, 2010/2011).

### Characterization of ciliate and endosymbiont

Characterization of the ciliate host and its bacterial endosymbiont was performed through the “full cycle rRNA approach”  (Amann, Ludwig & Schleifer, 1995)*,* i.e., through the nuclear 18S rRNA gene (generally considered the preferred marker to study molecular taxonomy and phylogeny of eukaryotic organisms—just as an example see Illa et al., 2019), ITS region and partial 28S gene (ciliate), and 16S rRNA gene (endosymbiont) sequencing, combined with Fluorescence *In Situ* Hybridization (FISH) experiments*.* Additionally, mitochondrial cytochrome c oxidase subunit I (COI) gene was sequenced as well, to display haplogroup variation in *P. multimicronucleatum* (Barth et al., 2006).

#### Molecular characterization

For molecular characterization of both ciliate host and bacterial endosymbiont, total DNA was extracted from approximately 50 *Paramecium* cells as described in Szokoli et al. (2016a) and Szokoli et al. (2016b). All PCR reactions were carried out with the Takara ExTaq (Takara, Kusatsu, Japan) reaction kit. COI gene was amplified using the forward primer F338dT and reverse primer R1184dT (Strüder-Kypke & Lynn, 2010), while for 18S rRNA gene amplification the forward 18S F9 Euk (Medlin et al., 1988) and the reverse 18S R1513 Hypo primers (Petroni et al., 2002) were used. The ITS region (including partial 18S rRNA gene, ITS1, 5.8S rRNA gene, ITS2, and partial 28S rRNA gene) sequence was obtained by PCR with the forward 18S F919 and the reverse 28S R671 primers (modified according to Boscaro et al., 2012). The 16S rRNA gene of the bacterial symbiont was amplified with the *Alphaproteobacteria*-specific forward primer 16S Alpha F19b and the universal bacterial reverse primer 16S R1522a (Vannini et al., 2004). PCR products were purified by NucleoSpin gel and PCR cleanup kit (Macherey-Nagel GmbH Düren, Germany) and directly sequenced at GATC Biotech AG (Constance, Germany). Internal primers were used to sequence 16S rRNA gene (Vannini et al., 2004), 18S rRNA gene (Rosati et al., 2004) and ITS regions (Boscaro et al., 2012). The latter two sequences were then joined together, exploiting the partial overlap on the 18S rRNA gene sequences. For the COI gene sequencing, amplification primers were employed from both directions.

#### FISH experiments

Preliminary FISH experiments were performed using the bacterial universal probe EUB338 (Amann et al., 1990) labeled with fluorescein-isothiocyanate (FITC) in combination with the specifically designed probe Rick_697 (5′-TGTTCCTCCTAATATCTAAGAA-3′) labeled with Cy3 to verify the presence of endosymbiotic bacteria belonging to the family *Rickettsiaceae* (Vannini et al., 2014). Based on the obtained results, i.e., the presence of a single, coincident positive signal in the ciliate macronucleus and the newly characterized 16S rRNA gene sequence corresponding to “*Ca*. Trichorickettsia mobilis”, a second FISH experiment was carried out using a species-specific probe, i.e., the probe Trichorick_142 (5′-GTTTCCAAATGTTATTCCATAC-3′) (Vannini et al., 2014) in combination with the bacterial universal probe EUB338 (Amann et al., 1990). The experiments followed the protocol by Fokin et al. (2019) employing a hybridization buffer containing no formamide, according to the recommendations for the used probes. Slides were mounted with SlowFade Gold Antifade with DAPI (Invitrogen, Carlsbad, USA) and viewed with a Zeiss AxioPlan fluorescence microscope (Carl Zeiss, Oberkochen, Germany) equipped with an HBO 100W/2 mercuric vapor lamp. Digital images were captured by means of a dedicated software (ACT2U, version 1.0).

### Planarian culturing

Planarians used in this work belonged to the species *Dugesia japonica*, asexual strain GI (Rossi et al., 2018). Animals were kept in a solution of: CaCl_2_ 2.5 mM, MgSO_4_ 0.4 mM, NaHCO_3_ 0.8 mM, and KCl 77µM, hereafter referred to as culturing water, at 18 °C in dim light conditions and fed with chicken liver (purchased from local food stores) prepared according to King & Newmark (2013), once a week. Non-regenerating specimens, within 5–8 mm of length, were used for all experimental procedures, after being starved for about two weeks.

### Transmission electron microscopy (TEM)

TEM preparations were obtained both for *P. multimicronucleatum* cells and planarians to study the ultrastructure of the ciliate-associated endosymbiont “*Ca.* Trichorickettsia mobilis*”* and to verify the success of transfection experiments (i.e., to check the animals for the presence of transferred ciliate endosymbionts in their tissues with particular attention to bacteria showing possible signs of division) respectively.

TEM preparations of *P. multimicronucleatum* cells were obtained according to Modeo et al. (2013b). Briefly, cells were fixed in a 1:1 mixture of 2.5% (v/v) glutaraldehyde in 0.1 M cacodylate buffer (pH 7.4), and 2% (w/v) OsO_4_ in distilled water, ethanol and acetone dehydrated, and embedded in Epon-Araldite mixture. Ultrathin sections were stained with 4% (w/v) uranyl acetate followed by 0.2% (w/v) lead citrate.

TEM preparations of planarian specimens were obtained as previously described (Rossi et al., 2014; Cassella et al., 2017), with minor modifications. Planarians were fixed with 3% glutaraldehyde in 0.1 M cacodylate buffer, and post-fixed with 2% osmium tetroxide for 2 h. After ethanol dehydration, samples were embedded in Epon-Araldite mixture. Ultrathin sections were stained with uranyl acetate and lead citrate and observed with a Jeol 100 SX Transmission Electron Microscope.

#### Negative staining

For negative staining, several *P. multimicronucleatum* cells of the strain US_Bl 16I1 were washed in distilled water and squashed; a drop of the resulting suspension was placed on a grid. Bacteria were allowed to settle for 2–3 min, then a drop of 1% uranyl acetate in distilled water was added for no longer than 1 min. The liquid was then absorbed with filter paper, the grid was air-dried, and observed under TEM (adapted from Sabaneyeva et al., 2018).

### Transfection experiments

Two transfection experiments with the same protocol were sequentially carried out over a period of four months. Each experiment was conducted by treating a fixed number of planarians with ciliate-enriched food, i.e., liver paste mixed with *P. multimicronucleatum* homogenate; from now on these animals will be referred to as “treated planarians”. As for experimental control, planarians fed plain liver paste were used; these animals will be referred to as “control planarians”. Each transfection experiment was performed according to the following protocol:

1. 96 planarians were selected by eye from culture (see above), washed in fresh culturing water and collected in a Petri dish with 50 µg/ml gentamicin sulfate (Sigma, Saint Luis, MO, USA) dissolved in their culturing water (see above). This preliminary antibiotic treatment was performed to minimize potential endogenous bacteria contaminants and was not harmful to the animals (Roberts-Galbraith, Brubacher & Newmark, 2016). Planarians were left in antibiotic treatment for 24 h under regular culturing conditions concerning temperature and light (see above) and kept under visual control during that period to verify their viability during the antibiotic treatment and by the time of transfer. Then, planarians were washed six times in their fresh culturing water, and split into two equal groups of 48 individuals each and accommodated in two different Petri dishes for the transfection procedure by means of feeding.
2. In order to get a bacterial load consistent with previous experiments (Abnave et al., 2014), 1.5 L of *P. multimicronucleatum* mass culture (cell concentration: ∼4 × 10^5^ cell/L) were selected. Indeed, considering that FISH experiments indicated roughly 100 endosymbionts/cell (see Results section), in total ciliates were estimated to contain ∼6 × 10^7^ symbiont cells. *P. multimicronucleatum* cells were filtered with a nylon filter (pore size: 100 µm), washed twice in sterile San Benedetto mineral water to minimize potential bacterial contaminants, concentrated and harvested by means of centrifugation (400× g per 10 min), so to reduce the medium volume to 2 ml. Then, cells were mechanically squashed and homogenized by syringing (syringe needle: 22GA, 0.70 mm in diameter) for 20 min at room temperature (adapted from Vannini et al., 2017). Cell homogenate was checked under the stereomicroscope so to exclude the presence of intact *Paramecium* cells, centrifuged (10,000× g per 10 min), and the resulting pellet was resuspended in 50 µl of planarian food (homogenized liver paste) by direct resuspension.
3. A group of 48 planarians was fed *P. multimicronucleatum*-enriched liver paste (treated planarians), while the other group was in parallel fed plain liver paste (control planarians). For feeding, food was spread over the bottom of the Petri dish. Animals were allowed to feed freely for a period of 2 h under regular culturing conditions (see above). Attention was paid to planarian survival and feeding behavior during this period. Two washing steps were then carried out removing the medium and adding fresh planarian culturing water. Finally, the two groups of animals were left in their fresh culturing water and in regular cultivation conditions in the two Petri dishes until the collection of specimens for the next TEM and PCR analyses at the three timepoints (see below) of the experiments. Animals were kept under visual control throughout the experiment to regularly check their viability.
4. At day 1, 3, and 7 after feeding (experimental timepoints), from each of the two Petri dishes containing treated and control planarians a group consisting of 16 animals were sampled: 4 animals were fixed and processed for TEM, and 12 were immediately frozen and stored at −80 °C, and dedicated to DNA extraction (4 animals per each sample).

### PCR verification of transfer success

Treated and control planarians were processed for genomic DNA extraction, purification, and PCR amplification with endosymbiont-specific primers. For each experimental condition (treated and control planarians) and each experimental timepoint (1, 3, and 7 days after feeding), genomic DNA was extracted from frozen samples by using the Wizard Genomics DNA purification kit (Promega, Madison, WI, USA). One microliter of purified DNA was analysed by gel electrophoresis to check for integrity. DNA was quantified using a Nanodrop spectrophotometer. For each experimental timepoint of both experimental conditions (i.e., treated and control planarians), similar amounts of genomic DNA were used for amplification using the ampli-Taq-gold DNA polymerase (Applied Biosystems, Foster City, CA, USA) and for a nested PCR assay. The first primer pair used, specific for “*Ca.* Trichorickettsia mobilis”, was RickFla_ F69 5′-GTTAACTTAGGGCTTGCTC-3′and Rick_R1455 5′-CCGTGGTTGGCTGCCT-3′ (Vannini et al., 2014); PCR conditions were: 3 min at 94 °C; 5 cycles consisting of 30 s at 94 °C, 30 s at 58 °C, 2 min at 72 °C each; 10 cycles consisting of 30 s at 94 °C, 30 s at 54 °C, 2 min at 72 °C each; 25 cycles consisting of 30 s at 94 °C, 30 s at 50 °C, 2 min at 72 °C each; ending with 7 min at 72 °C. The second, nested primer pair used was RickFla_F87 5′-CTCTAGGTTAATCAGTAGCAA-3′and Rick_R1270 5′-TTTTAGGGATTTGCTCCACG-3′ (Vannini et al., 2014). For nested PCR assay, one microliter of PCR product of the first PCR assay was used as a template; PCR conditions were as above.

For each experimental condition and each timepoint of the two transfection experiments, the DNA amplification was performed in duplicate. Samples were considered positive if a single band of the expected size was recorded after nested amplification. Sequencing of amplicons was carried out to confirm the presence of “*Ca.* Trichorickettsia mobilis” using the primer RickFla_F87 (see above) and Sanger sequencing (BMR Genomics, Padova, Italy).

Samples obtained from planarians fed with plain liver paste were used as control. As positive control, genomic DNA purified from *P. multimicronucleatum* monoclonal strain US_Bl 16I1 was processed.

## Results

### Host morphological and molecular identification

**Figure 1:**
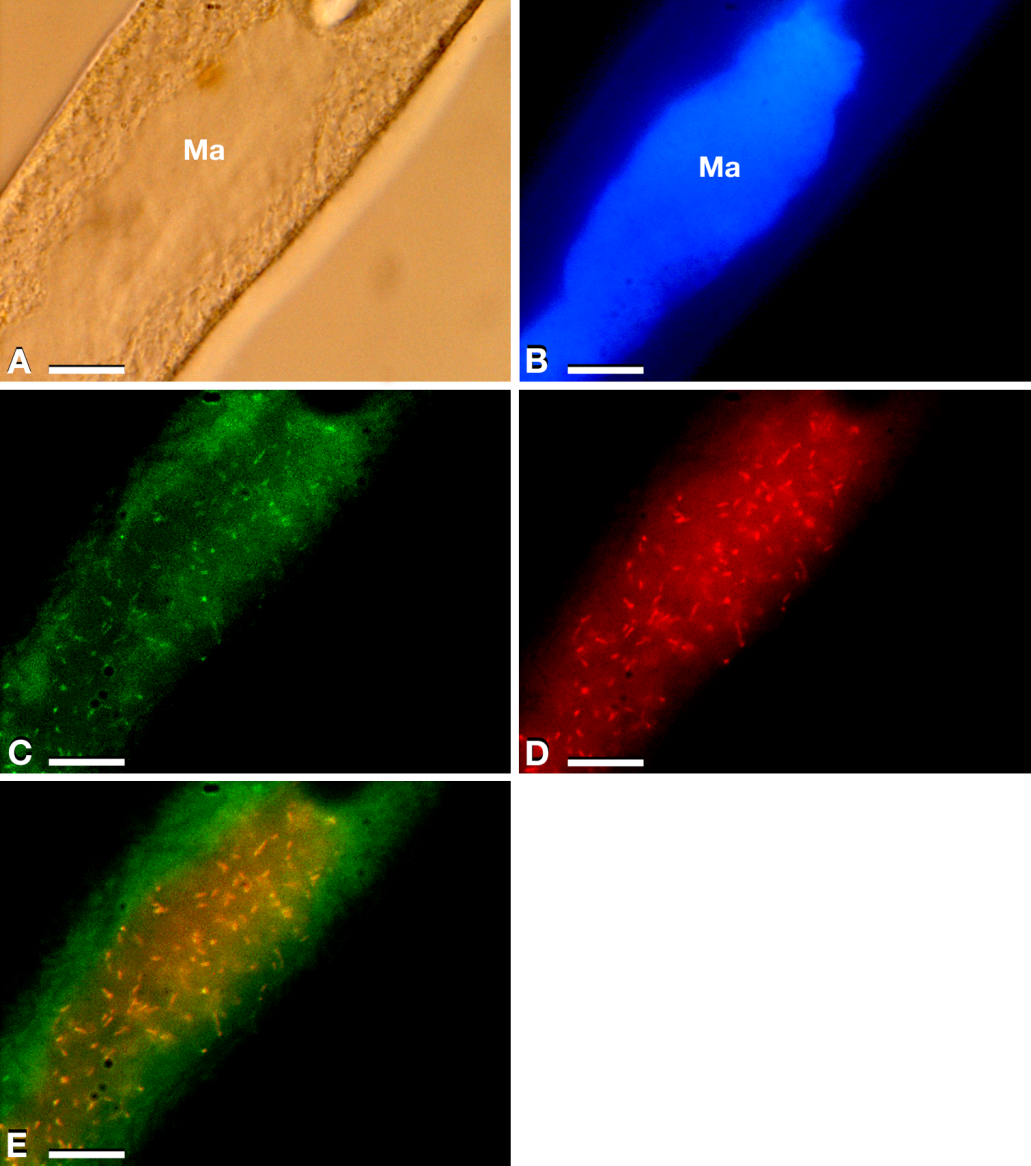
Results of FISH experiments on a *P. multimicronucleatum* US_Bl 16I1 cell. (A) Bright field microscopy (Ma, macronucleus). (B) After DAPI staining. (C) Treated with the almost universal bacterial probe EUB338 double labelled with fluorescein (green) (probe labelling both at 5′ and 3′ ends). (D) Treated with species-specific probe Trichorick_142 labelled with Cy3 (red) targeting “*Ca.* Trichorickettsia mobilis”. (E) Merge of C and D. The number of endosymbionts targeted by the species-specific probe in the macronucleus reached ∼100 (D, E). Scale bars stand for 10 µm.

Ciliate strain US_Bl 16I1 (Figs. 1A, 1B; Figs. S1 and S2 in Article S1) was confirmed in morphological inspections as *Paramecium multimicronucleatum* Powers & Mitchell, 1910 considering features of key-characters such as cell size, number and features of micronuclei (mi), and number and features of contractile vacuoles (Figs. S1 and S2 in Article S1), as described in previous literature (Wichterman, 1986; Allen, 1988; Fokin, 1997; Fokin & Chivilev, 2000; Fokin, 2010/2011). The molecular analysis of the combined (partial) 18S rRNA-ITS1-5S rRNA gene-ITS2-(partial) 28S rRNA gene sequence (2,792 bp, GenBank accession number: MK595741) confirmed the species assignation by morphological identification with a 100% sequence identity with the sequences of other *P. multimicronucleatum* strains already present in GenBank presenting either 18S rRNA gene portion only (AB252006 and AF255361), or the ITS portion (AY833383, KF287719, JF741240 and JF741241). COI gene sequence identity of strain US_Bl 16I1 (760 bp, accession number: MK806287) is highest with another *P. multimicronucleatum* haplotype (FJ905144.1; 96.3%).

### Endosymbiont identification and features

The 16S rRNA gene amplification was performed to identify endosymbionts associated with *P. multimicronuclatum* US_Bl 16I1 strain (sequence length 1,563 bp, accession number: MK598854), which allowed to assign it to “*Ca.* Trichorickettsia mobilis”. Specifically, among the three subspecies previously described, it was more similar to “*Ca.* Trichorickettsia mobilis subsp. hyperinfectiva”, an endosymbiont described in the cytoplasm of *Paramecium calkinsi* (99.8% identity of the novel sequence with type strain MF039744.1) (Sabaneyeva et al., 2018). FISH experiments (Figs. 1C–1E) performed by using bacterial universal probe EUB338 (Fig. 1C) and the species-specific probe Trichorick_142 (Fig. 1D) confirmed this result and indicated that, contrarily to the *P. calkinsi* symbiont, the bacterium was localized in the ciliate macronucleus (roughly with a presence of about 100 endosymbionts per cell). Additionally, the full overlapping of bacterial universal probe EUB338 and “*Ca*. Trichorickettsia”-specific probe signals indicated that this symbiont constituted the total set of intracellular bacteria in host *P. multimicronucleatum* US_Bl 16I1 cells (Fig. 1E, merge).

**Figure 2:**
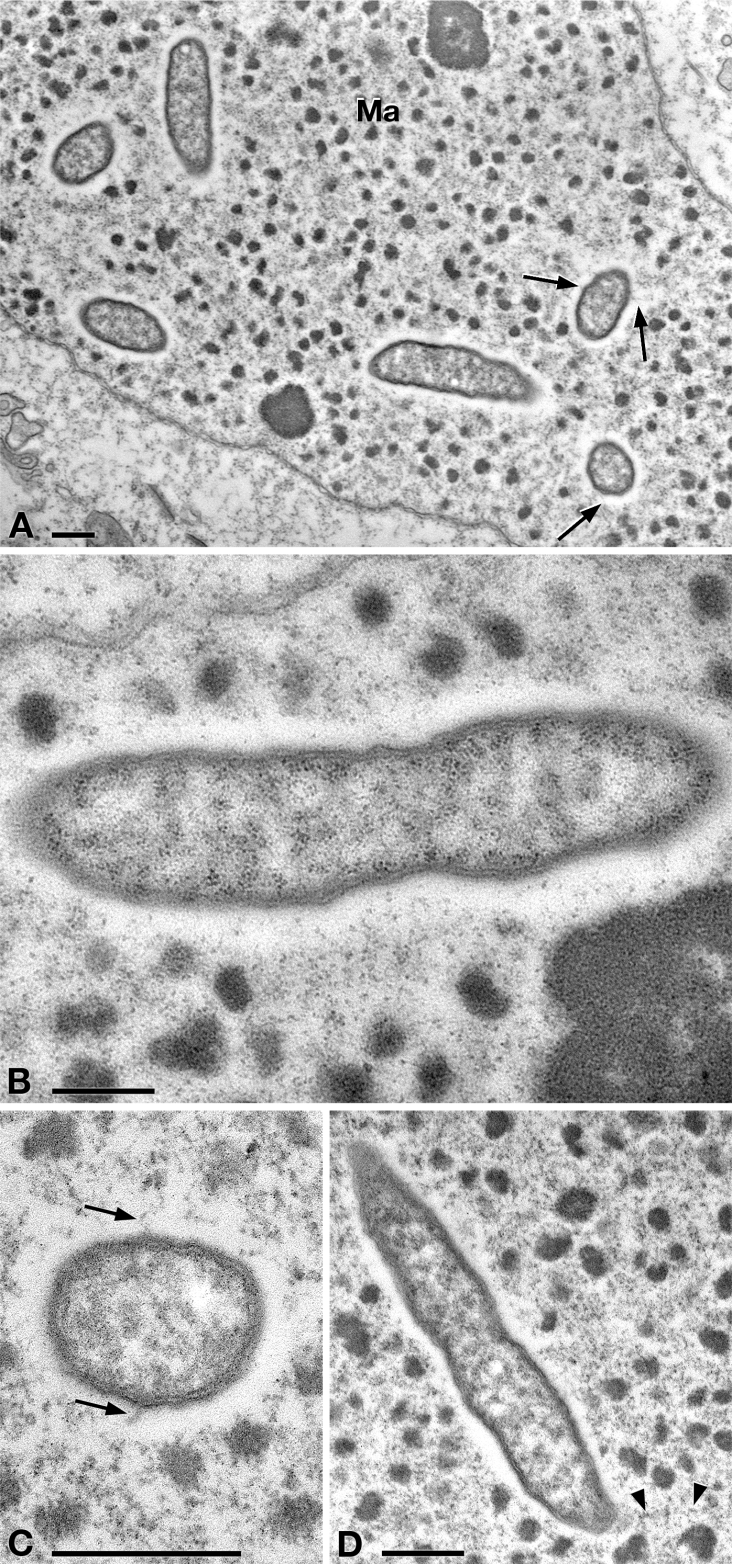
TEM pictures showing the morphological-ultrastructural details of “*Ca.* Trichorickettsia mobilis” in the macronucleus of *P. multimicronucleatum* US_Bl 16I1. (A) Longitudinally and transversely sectioned endosymbionts inside macronucleus (Ma) with a double membrane and surrounded by a clear halo; electron-lucid roundish areas occur in the cytoplasm of some endosymbionts. (B) Numerous bacterial ribosomes are visible. (C, D) Bacterial flagella are short, located either around (arrow) the endosymbiont cell or at a cell pole, where they can form a putative tail (arrowhead). Scale bars stand for 0.5 µm.

**Figure 3:**
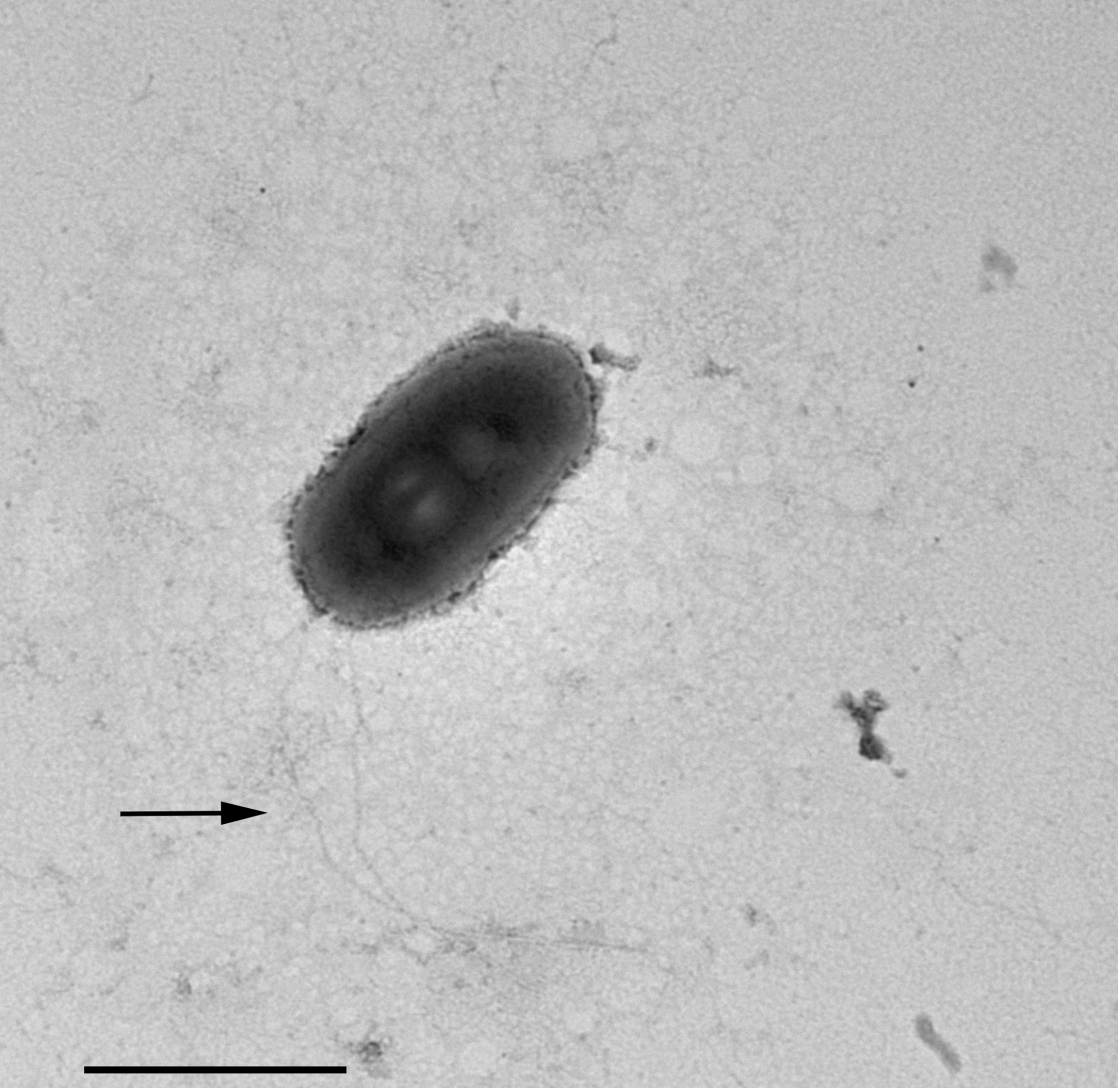
Negative contrast at TEM of a specimen of “*Ca.* Trichorickettsia mobilis”. Some short flagella are present and at least two longer flagella (arrow) arise from one of cell ends. Scale bar stands for 1 µm.

In TEM-processed ciliate cells, the endosymbionts were confirmed to be hosted in the macronucleus; they showed a two-membrane cell wall typical of Gram-negative bacteria and appeared rod-shaped, with rounded to narrower ends (Fig. 2). They were surrounded by a clear halo and not encircled by any symbiosomal vacuole, i.e., they appeared in direct contact with host nuclear material. Their size was ∼1.2–2.1 × 0.5–0.6 µm, and they were scattered throughout the macronucleus of the ciliate (Figs. 2A–2D). Sometimes in their cytoplasm several electron-lucid “holes” (diameter: ∼0.2 µm), were observed (Fig. 2A). Bacterial cytoplasm was electrondense; no other structures were visible except for abundant ribosomes (Fig. 2B). Endosymbionts displayed thin (diameter: ∼9 nm) and short flagella distributed all around the cell (Figs. 2A, 2C–2D) which sometimes formed a putative tail emerging from a cell end (Fig. 2D). The presence of longer flagella was evident after negative staining procedure (Fig. 3): besides some short flagella occurring all around the cell, at least a few longer and thicker flagella (∼2000 × 12 nm) arose from one of cell ends.

Some active bacterial motility, likely obtained by means of their flagella, was recorded inside the macronucleus of intact *P. multimicronucleatum* cells during observation under DIC microscope at 1,000×. Endosymbionts were seen to move through the chromatin bodies covering short distances; additionally, after ciliate cell squashing, the released bacteria were still capable of movement (L Modeo, pers. obs., 2017).

### Transfection experiments

To verify RLO transfer to planarians, we fed animals either with ciliate-enriched liver paste (treated planarians) or plain liver paste (control planarians). 100% survival of the planarians (control and treated animals) was confirmed throughout the transfection experiments; in particular, the survival of treated planarians was not affected by the treatment and the animals did not show any morphological or behavioral alteration. The success of each of the two independent transfection experiments performed was investigated by means of PCR experiments and TEM observation on treated and control planarians at the different experimental timepoints, according to the described experimental procedure.

**Figure 4:**
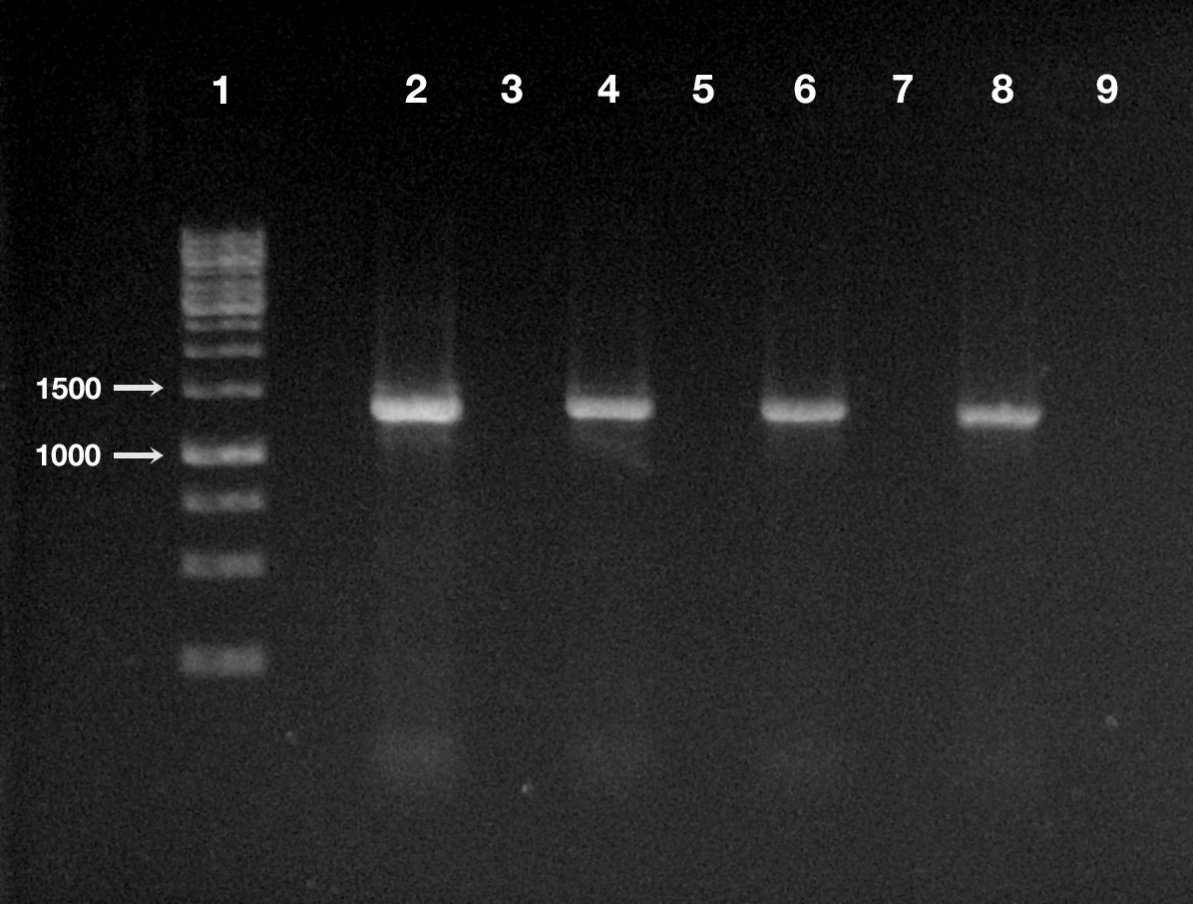
Detection of “*Ca.* Trichorickettsia mobilis” DNA in treated and control planarians by PCR. Line 1, ladder (Gene Ruler 1 Kb DNA ladder Fermentas, Waltham, Massachusetts, USA); line 2, positive control (i.e., DNA extracted from *P. multimicronucleatum* US_Bl 16I1); line 3, negative control (i.e., no DNA); line 4, treated planarian at day 1 after feeding (a.f.); line 5, control planarian at day 1 a.f.; line 6, treated planarian at day 3 a.f.; line 7, control planarian at day 3 a.f.; line 8, treated planarian at day 7 a.f.; line 9, control planarian at day 7 a.f.

The DNA of “*Ca.* Trichorickettsia mobilis” was detected at all experimental timepoints (day 1, 3, and 7 after feeding) in genomic DNA preparations obtained from all investigated planarians fed with *P. multimicronucleatum* lysate-enriched liver paste. The size of the nested amplicon (1,360 bp) obtained from treated planarian samples matched the size of the amplicon obtained in DNA purified from ciliate monoclonal strain US_Bl 16I1 (positive control) (Fig. 4). The sequencing of the obtained amplicons confirmed the specificity for “*Ca*. Trichorickettsia mobilis” (100% sequence identity with the sequenced obtained from the symbiont). No amplification product was obtained in genomic DNA samples from control planarians fed with plain liver paste (negative control) (Fig. 4).

Ultrastructural observation was conducted on all TEM-processed specimens per each timepoints of the experiments, for both experimental groups, i.e., treated and control planarians.

**Figure 5:**
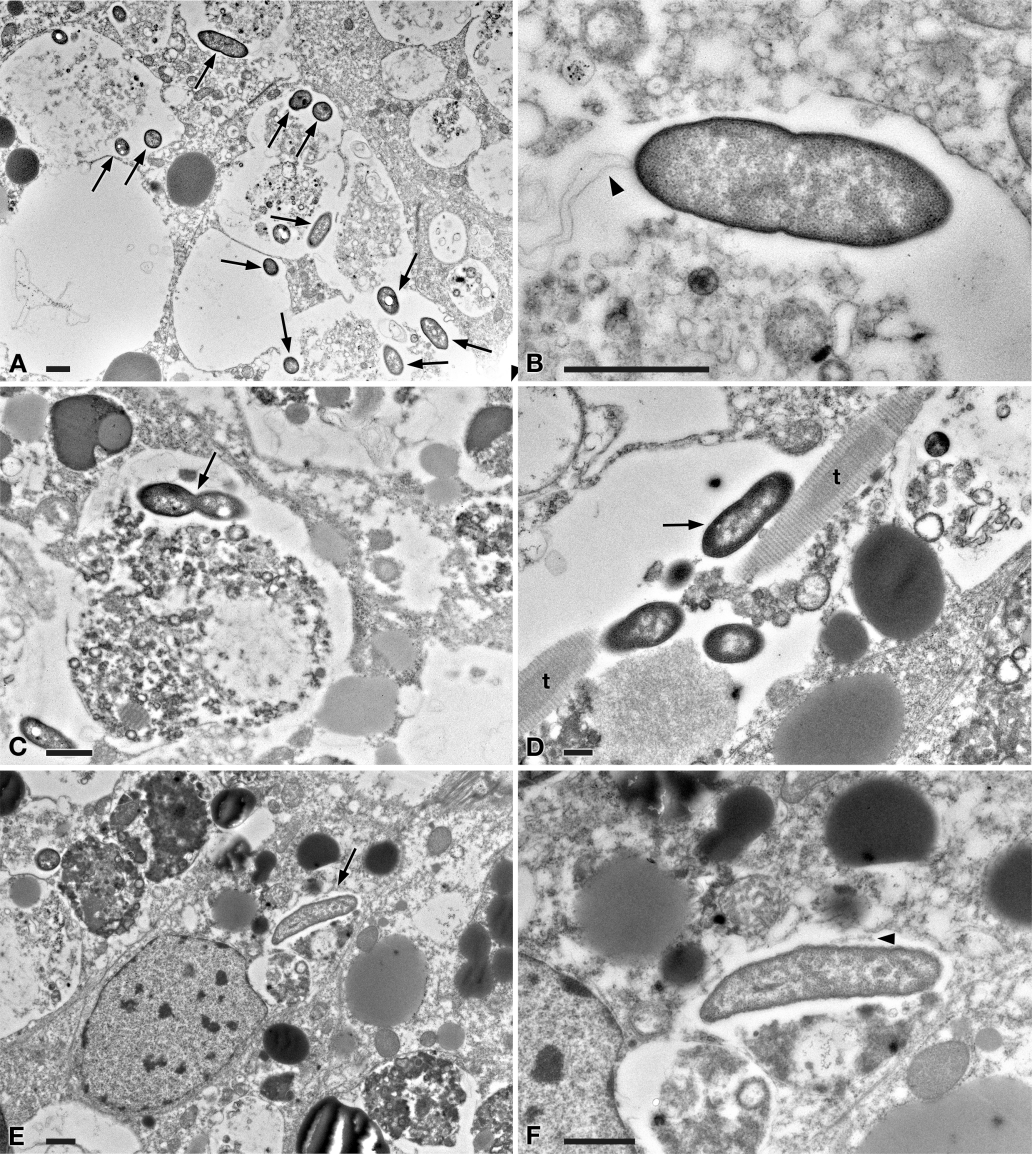
TEM pictures of the intestine of antibiotic-treated *D. japonica* fed on liver paste enriched with the homogenate of *P. multimicronucleatum* US_Bl 16I1 cells and fixed at day 1 after feeding. (A) A view through planarian intestine where several bacteria are visible (arrows). (B) Enlargement of a particular of (A) to show a dividing bacterium with flagella arising from its pole (arrowhead). (C) A dividing bacterium (arrow) in planarian intestine (D) Extruded trichocysts (t) (extrusive organelles) of *Paramecium* detected in planarian intestine along with bacteria, also dividing (arrow). (E) A longitudinally sectioned bacterium (arrow) free in the cytoplasm of an intestinal cell of a treated planaria. (F) Enlargement of a particular of (E). Arrowhead, flagella. Scale bars stand for 1 µm.

**Figure 6:**
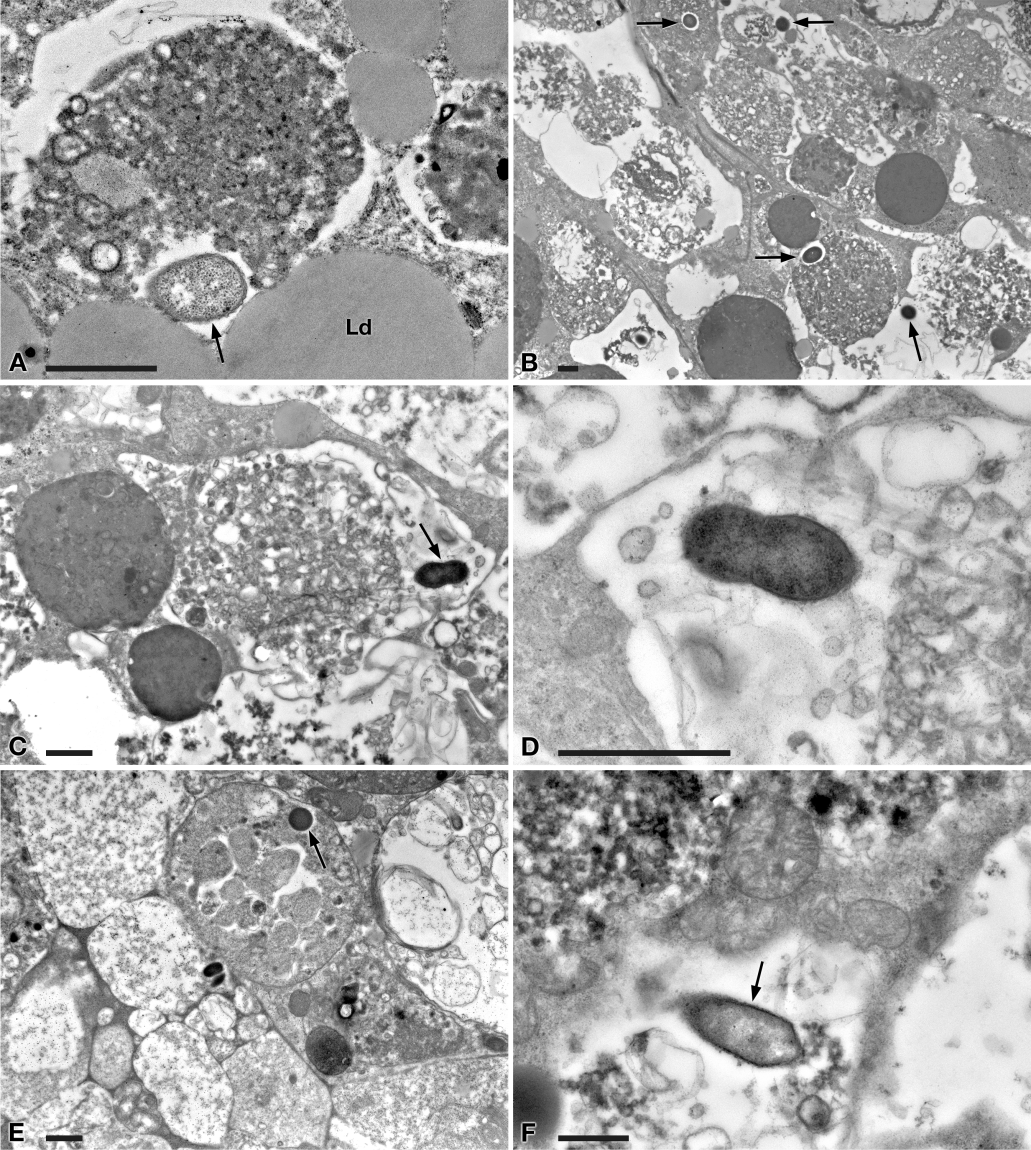
TEM pictures of the intestine of antibiotic-treated *D. japonica* fed on liver paste enriched with the homogenate of *P. multimicronucleatum* US_Bl 16I1 cells and fixed at day 3 and 7 after feeding. (A–D) day 3 after feeding, (E–F) day 7 after feeding. (A, B) A bacterium (arrow) in proximity with the border of a phagosome, in the cytoplasm of a planarian intestinal cell. Ld, lipidic droplet. (C) A dividing bacterium** (**arrow**)** next to a phagosome. (**D**) Enlargement of a particular of (**C**). (**E**) Bacteria (arrow) inside phagosome and (**F**) free in the cytoplasm. Scale bars stand for 1 µm.

In tissues collected from all the investigated planarians fed with *Paramecium* US_Bl 16I1 lysate-enriched liver paste, the presence of bacteria with morphology and sizes (see below for details) fitting with those of ciliate endosymbionts within intestinal cells was detected at all timepoints of the two experiments (Figs. 5 and 6).

At day 1 after feeding, several bacteria were recognizable in planarian enterocytes. Some of them appeared to be in division (Figs. 5A–5D). In several cases, the membrane of bacteria-including phagosomes was damaged and interrupted, and bacteria showing a two-membrane cell wall and a surrounding clear halo were detected free in the cytoplasm of planarian intestinal cells (i.e., in direct contact with their cytoplasm) (Figs. 5E, 5F). Flagella (diameter: ∼9 nm) could also be detected on bacterial surface (Figs. 5B, 5F). Additionally, extruded trichocysts (extrusive organelles typical of *Paramecium*), presumably included in *Paramecium* homogenate and ingested by treated planarians, were easily recognizable inside intestinal cell phagosomes (Fig. 5D).

A similar scenario was observed at day 3 after feeding (Figs. 6A–6D), with several bacteria in apparent division as well (Figs. 6C, 6D). At day 7 after feeding, a few bacteria (i.e., roughly less bacteria than the several observed after 1 and 3 days) were still well visible in planarian enterocytes (Fig. 6E), also free in the cytoplasm (Fig. 6F).

No bacteria were ever observed in tissues other than intestine in treated planarians; similarly, no bacteria were observed in tissues of TEM-processed control animals (Fig. S3).

## Discussion

A “*Ca*. Trichorickettsia mobilis” endosymbiont, closely related to “*Ca.* Trichorickettsia mobilis subsp. hyperinfectiva” previously described in the cytoplasm of *P. calkinsi* (Sabaneyeva et al., 2018), was retrieved in the macronucleus of the ciliate *P. multimicronucleatum* strain US_Bl 16I1. “*Ca.* Trichorickettsia mobilis” up to now has been exclusively found as an endosymbiont of ciliates belonging to the genera *Paramecium* and *Euplotes* (Vannini et al., 2014; Sabaneyeva et al., 2018). In the light of the present findings, a comparison among all strains characterized to date suggesting a certain morphological plasticity of this bacterium, is presented in Article S1.

The aim of the present research was to obtain a first indication on the potential transfer of the *Rickettsia*-related macronuclear endosymbiont “*Ca.* Trichorickettsia mobilis” from the ciliate *P. multimicronucleatum* strain US_Bl 16I1 to the metazoan model *D. japonica*. There are several studies reporting on the host/symbiont relationships of different *Paramecium* species with different *Rickettsiales*; *P. multimicronucleatum* lies in this ciliate selection, and is a rather a common species, sharing the freshwater habitat with planarians. Thus, it was chosen as donor in transfection experimental context as, in our opinion, it can be considered a suitable candidate as putative environmental vector for RLOs. As we dealt with endosymbionts, which are present in limited numbers inside their ciliate host, we chose to treat the planarians with cell mass culture homogenate instead of adding living ciliates to planarians food. This allowed processing of as many ciliates as possible to maximize the probability of endosymbiont ingestion by the animals, thereby increasing the chance of detection of successful transfer via PCR and TEM-based approaches.

According to the present findings, the transfection experiments were successful, i.e., they showed the capability of “*Ca.* Trichorickettsia mobilis” to enter planarian tissues. Indeed, in the intestine of planarians previously antibiotic-treated to avoid bacterial contamination and fed with liver paste enriched with pellet of ciliate homogenate (rich in “*Ca*. Trichorickettsia” symbionts), “*Ca*. Trichorickettsia” bacterial cells in apparent division were observed up to day 3 after feeding. We observed several bacteria free from phagosomal membrane, i.e., in direct contact with enterocyte cytoplasm (Figs. 5 and 6). The presence of bacteria with a morphology and a size fully resembling those of the RLO endosymbiont of *P. multimicronucleatum* US_Bl 16I1 were observed up to day 7 after feeding inside and outside planarian phagosomes (Fig. 6). Based on our findings, we hypothesize that dividing bacteria might also be present in treated planarian parenchyma after day 3.

On the contrary, with respect to treated animals, in TEM preparations of controls (i.e., antibiotic-treated planarians fed with plain liver paste) no bacteria were found (Fig. S3).

These results were confirmed and supported by PCR analysis and sequencing of obtained amplicons: the DNA of “*Ca.* Trichorickettsia mobilis” was recovered in treated planarians up to day 7 after feeding while in control animals no RLO DNA was ever amplified and detected (Fig. 4). On the other side, survival of treated planarians was not affected by the treatment and animals did not manifest morphological or behavioral alterations.

Our findings are in line with those by Gong et al. (2016). These authors studied the phylogenetic identities of digestion-resistant bacteria that could survive starvation and form relatively stable associations with some marine and freshwater ciliate species, and demonstrated that the classes *Alphaproteobacteria* (which includes the order *Rickettsiales*) and *Gammaproteobacteria* are prevalent as digestion-resistant bacteria; from this study a putative significant role of secretion systems in promoting marine protist-bacteria associations resulted as well.

In our experiments, after being ingested by planarians, the bacteria were observed enclosed inside phagosomes of intestinal cells. This also occurred in previous experiments investigating the resistance of planarians to infection by bacterial strains pathogenic for humans and other metazoans (Abnave et al., 2014). In that research, planarians could eliminate most of the phagocytized bacterial strains within 3–6 days post-feeding thanks to 18 resistance genes, such as *MORN2*, so the authors suggested that planarians can be considered a model to identify innate resistance mechanisms. Under this perspective, the evidence we obtained that the “*Ca*. Trichorickettsia” endosymbionts of *P. multimicronucleatum* US_Bl 16I1 are still detectable in planarian intestine enterocytes inside and outside phagosomes up to day 7 after feeding is a good indication that the bacteria are able to survive, at least temporarily, within this time-span. This is reinforced also considering that the bacterial morphology in the planarians is comparable to that of endosymbionts in their original localization inside the macronucleus of *P. multimicronucleatum* (Figs. 2, 5 and 6), thus not evidencing any lethal alteration. Along this line of thought, we hypothesize that “*Ca.* Trichorickettsia mobilis” might be capable to avoid typical defense mechanisms exploited by planarians entering planarian tissues and transiently surviving within. Interestingly, similarly to some *Gammaproteobacteria* such as *Rheinheimera* sp. strain EpRS3 (*Chromatiaceae*), capable of escaping from phagosomes of the ciliate *Euplotes aediculatus* when fed the bacterium plus its culture medium (Chiellini et al., 2019), *Rickettsiaceae* are already known for their ability to escape the host vacuolar membrane, residing freely in the host cytoplasm, where they may exploit host cytoskeleton for movement (Renesto et al., 2003; Gouin et al., 2004; Walker & Ismail, 2008; Cardwell & Martinez, 2009; Ge & Rikihisa, 2011; Rennoll-Bankert et al., 2016).

In the past, efforts have been put to experimentally verify the transfer of morphologically RLOs among aquatic organisms. For example, Nunan et al. (2003) performed bioassays to verify the transfer of the infection between two species of commercially farmed shrimps, i.e., the infected *Penaeus monodon* and the specific pathogen-free *Penaeus vannamei*, with the aim of investigating the suspected causative agent of severe mortality in farms where those organisms are in co-culture (grow-out ponds). In that case, only injection bioassays were successful leading to an infection, while per *os* infection failed. Among different possible reasons for this negative result those authors cited the potential need for a vector to disseminate the disease. According to our findings, ciliates could be seen as suitable vectors in this kind of situation.

## Conclusions

To the best of our knowledge, this is the first time that a set of experimental bioassays was performed to investigate the possible transfer of a “true” and ascertained *Rickettsiales* bacterium from an infected protist to an uninfected metazoan of the same aquatic environment (freshwater). We believe that our findings, indicating, at least transiently, an effective transfer of ciliate endosymbionts to the intestinal cells of planarian, can offer intriguing insights concerning the diseases caused by *Rickettsiales* or RLOs occurring in fish farms or in the wild. Indeed, our study reinforces the notion that these might have ciliates or other protists as putative vectors. Although further investigations on this topic are necessary to expand its implications, we think that our study may represent the basis for conceiving long-lasting experiments aiming to better understand whether “*Ca.* Trichorickettsia mobilis”, as well as other *Rickettsiales* symbionts of protists, can be able to survive longer in tissues of planarians and other aquatic Metazoa, and whether these RLOs may have some impact on the recipient host health.

##  Supplemental Information

10.7717/peerj.8977/supp-1 Data S1 Gene sequences obtained in the present work for *Paramecium multimicronucleatum* strain US_Bl 16I1 (COI and 18S_ITS_28S) and its endosymbiont ”*Ca.* Trichorickettsia mobilis” (16S) COI gene and combined (partial) 18S rRNA-ITS1-5S rRNA gene-ITS2-(partial) 28S rRNA sequences of *Paramecium multimicronucleatum* strain US_Bl 16I1: GenBank accession numbers MK595741 and MK806287 respectively. 16S rRNA gene sequence of “*Ca.* Trichorickettsia mobilis”: Gen Bank accession number MK598854.

10.7717/peerj.8977/supp-2 Data S2 In vivo measurements of length, width, and buccal cavity length of 10 *Paramecium multimicronucleatum* strain US_Bl 16I cells for species identification

10.7717/peerj.8977/supp-3 Figure S3 Trans-infection experiments: TEM observation the intestine of antibiotic-treated *D. japonica* fixed after feeding with plain liver paste (control planarians) (**A**) Day 1 after feeding. (**B**) Day 7 after feeding. L, lumen of planarian intestine. No bacteria were observed in tissues of investigated control animals in none of the experimental timepoints. Scale bars stand for 1µm.

10.7717/peerj.8977/supp-4 Supplemental Information 1 Morphological-ultrastructural characterization of *Paramecium multimicronucleatum* strain US_Bl 16I and discussion on the morphological plasticity of its endosymbiont ”*Ca.* Trichorickettsia mobilis”

## Additional Information and Declarations

### DNA Deposition

The following information was supplied regarding the deposition of DNA sequences: All the sequences are available in the Supplemental Files and at Genbank: - Combined (partial) 18S rRNA-ITS1-5S rRNA gene-ITS2-(partial) 28S rRNA gene sequence of *Paramecium multimicronucleatum* strain US_Bl 16I1: MK595741; - COI gene sequence identity of strain US_Bl 16I1: MK806287; - 16S rRNA gene sequence of the *Paramecium multimicronucleatum* US_Bl 16I1 endosymbiont “*Ca.* Trichorickettsia mobilis” US_Bl 16I1 strain: MK598854.

### Data Availability

The following information was supplied regarding data availability: The raw data are available in the Supplemental Files.
