# Supplementary material for: “Candidatus Trichorickettsia mobilis”, a Rickettsiales bacterium, can be transiently transferred from the unicellular eukaryote Paramecium to the planarian Dugesia japonica
Source: PeerJ. 2020 Apr 23;8:e8977. doi: 10.7717/peerj.8977 (PMC7183750; doi:10.7717/peerj.8977)
Supplement: Data S2 [file peerj-08-8977-s002.docx]

| Cell | Length (µm) | Width (µm) | Buccal cavity length (µm) |
| --- | --- | --- | --- |
| 1 | 249.0 | 84.5 | 45.4 |
| 2 | 227.3 | 63.3 | 44.9 |
| 3 | 231.8 | 63.4 | 54.5 |
| 4 | 227.4 | 68.0 | 45.4 |
| 5 | 226.8 | 59.1 | 50.0 |
| 6 | 227.3 | 63.6 | 40.9 |
| 7 | 218.7 | 67.1 | 32.8 |
| 8 | 250.0 | 85.4 | 45.0 |
| 9 | 239.0 | 82.5 | 41.6 |
| 10 | 297.0 | 93.7 | 53.5 |
