# Supplementary figures and images for: “Candidatus Trichorickettsia mobilis”, a Rickettsiales bacterium, can be transiently transferred from the unicellular eukaryote Paramecium to the planarian Dugesia japonica
Source: PeerJ. 2020 Apr 23;8:e8977. doi: 10.7717/peerj.8977 (PMC7183750; doi:10.7717/peerj.8977)

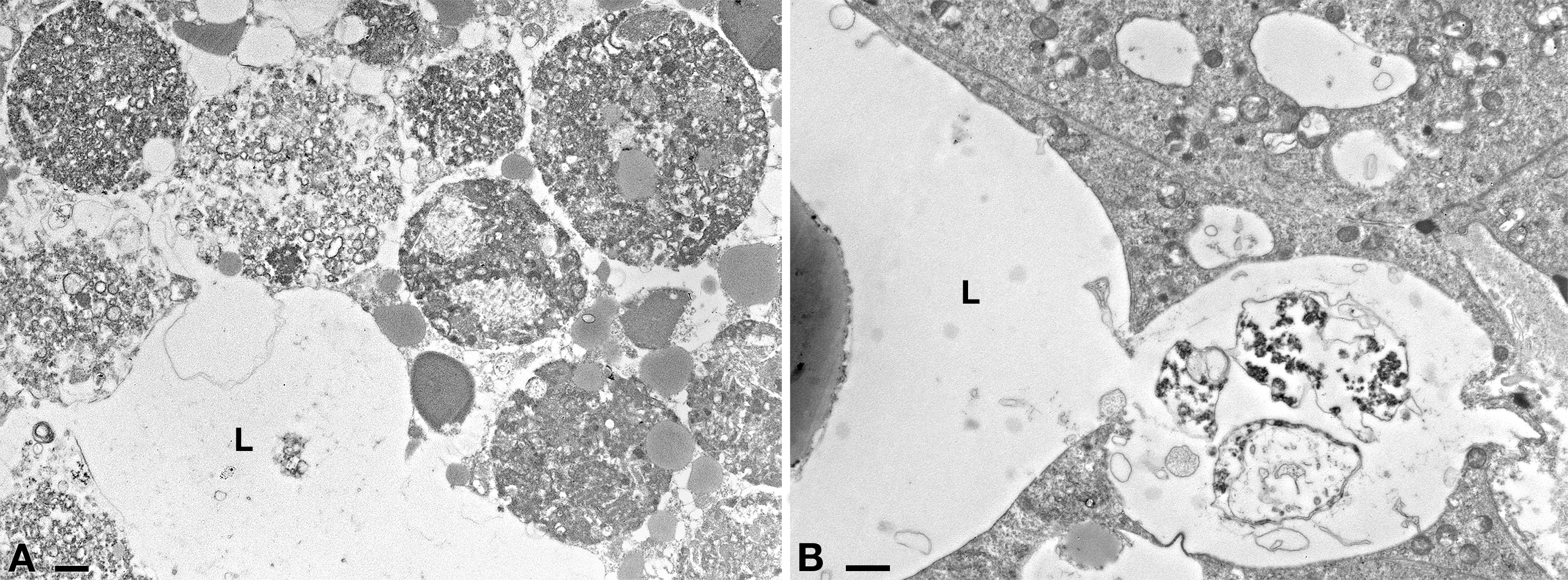

Supplement: Figure S3 — (A) Day 1 after feeding. (B) Day 7 after feeding. L, lumen of planarian intestine. No bacteria were observed in tissues of investigated control animals in none of the experimental timepoints. Scale bars stand for 1µm. [file peerj-08-8977-s003.png]
