# Supplementary material for: “Candidatus Trichorickettsia mobilis”, a Rickettsiales bacterium, can be transiently transferred from the unicellular eukaryote Paramecium to the planarian Dugesia japonica
Source: PeerJ. 2020 Apr 23;8:e8977. doi: 10.7717/peerj.8977 (PMC7183750; doi:10.7717/peerj.8977)
Supplement: Supplemental Information 1 [file peerj-08-8977-s004.pdf]

Morphological-ultrastructural characterization of *Paramecium multimicronucleatum* strain US_Bl 16I

Live observation (Fig. S1). *P. multimicronucleatum* strain US_Bl 16I1 showed elongated cells with a mean cell size of ~ 239 x 73 µm, with a buccal cavity length of ~ 45 µm (mean values; measurements taken on 10 specimens). Anterior cell end was rounded, posterior cell end was narrower; macronucleus was ellipsoidal (size ~ 100 x 50 µm) and situated near cell equator (Figs. S1A, S1B). Two contractile vacuole pores were visible near cell poles (Fig. S1B). Spindle trichocysts (the extrusive organelles typical of genus *Paramecium*) were observed inserted in the cortex (Fig. S1C). According to Feulgen stained cells, three spheroidal micronuclei of vesicular type were located in proximity of macronucleus (Fig. S1D).

Fine structure (Fig. S2). Spindle trichocysts were docked in the cortex (size: ~ 10 x 1.6 µm) near somatic ciliature and flanked by numerous, large mitochondria (Fig. S2A). Some almost extruding trichocysts were also visible (Fig. S2B). Slender, rod shaped, non-flagellated bacteria (size: ~ 2.0 x 0.3 µm), sometimes presenting more cytoplasmatic electron-dense regions, were included together with flagellates in large digestive vacuoles; macronucleus showed small chromatin bodies, larger, electron-dense nucleoli, and several scattered bacterial endosymbionts (Fig. S2C). Macronucleus appeared encircled by a layer of clearer, rarefied cytoplasm (~ 1.8 µm thick) devoid of organelles (Fig. S2D). Nuclear envelope pores were well visible (Fig. S2E). Micronuclei (~ 3 µm in length) showed a central vesicular mass encircled by an electron-transparent zone (Fig. S2F).

Figure S1. Light microscope observation of *P. multimicronucleatum* strain US_Bl 16I1. (A-C) *In vivo* specimens; (**D**) Feulgen stained cell. Ma, macronucleus. (**C**) the cytostome (c) at a higher magnification with trichocysts (t) inserted in the cortex. (**D**) Feulgen staining highlights the Ma and the three micronuclei (mi). Scale bars stand for 10 µm.

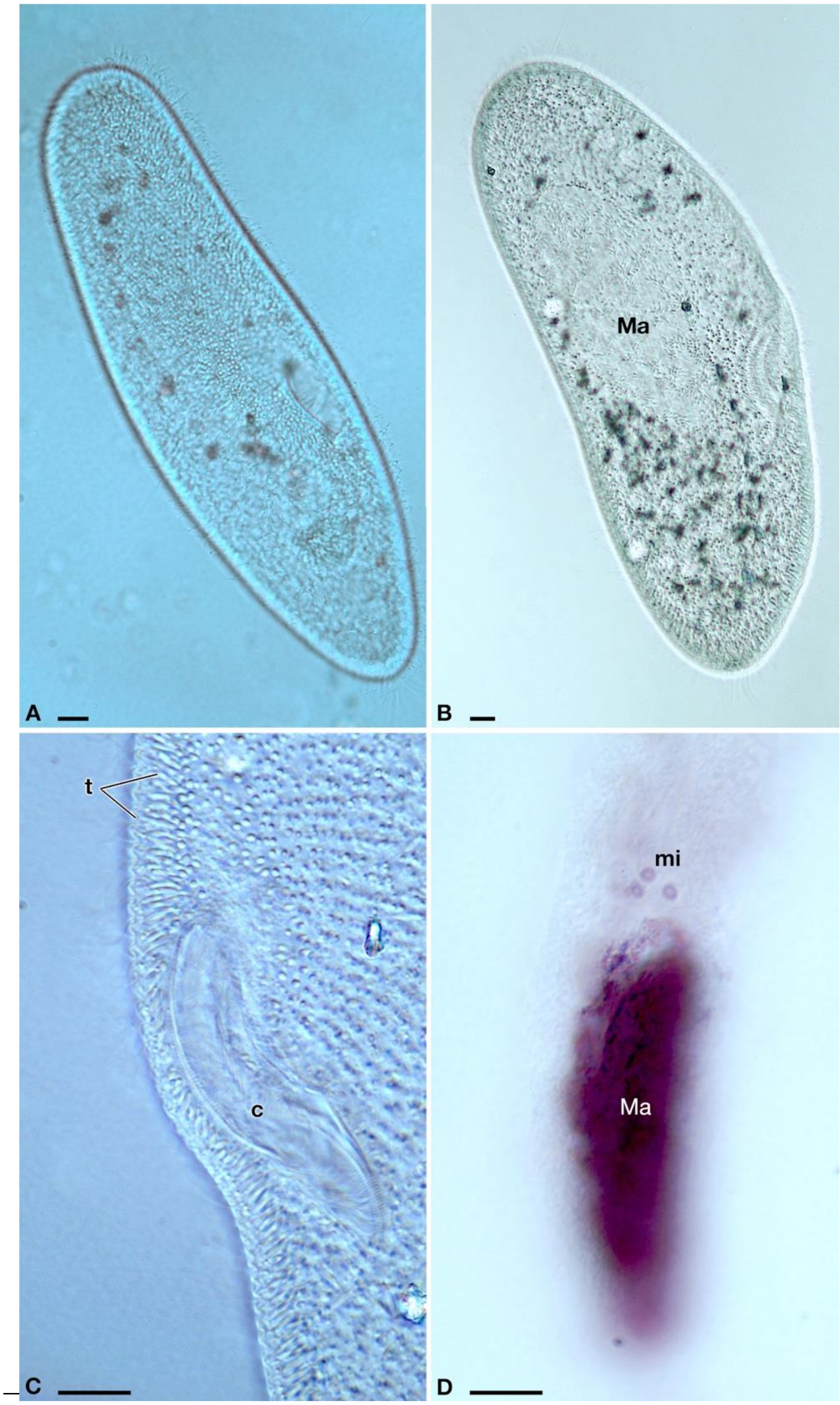

Figure S2. TEM pictures of *P. multimicronucleatum* strain US_Bl 16I1. (**A, B**) cortex with trichocysts inserted (t) in resting state (**A**) and about to extrude (**B**); m, mitochondria. (**C, D**) macronucleus (Ma) encircled by a layer of rarefied material (asterisk) with endosymbionts (arrows) and nucleoli (n); Ph, phagosomes. (**E**) Endosymbionts inside the Ma, a transversally sectioned T occurs near Ma membrane. (**F**) Ma portion and one of the three micronuclei (mi). Scale bars stand for 1 µm.

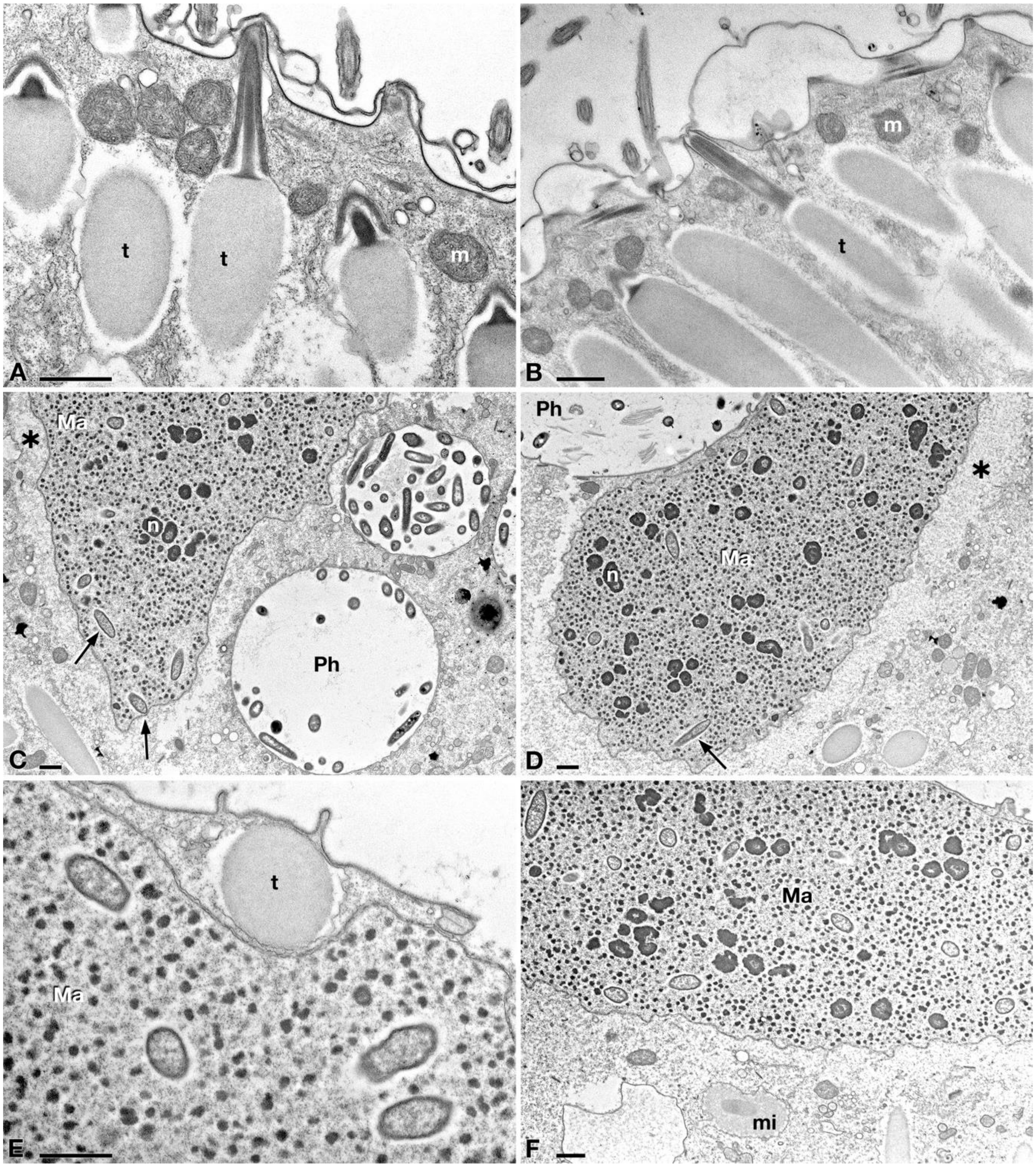

"*Candidatus* (*Ca.*) Trichorickettsia mobilis", endosymbiont of different ciliates, showing morphological plasticity.

The alphaproteobacterium "*Ca.* Trichorickettsia mobilis" has been retrieved only as endosymbiont of different ciliates [1]. At present, three subspecies have been identified on molecular basis [2], namely "*Ca.* Trichorickettsia mobilis subsp. mobilis", hosted in the macronucleus of *P. multimicronucleatum*, "*Ca.* Trichorickettsia mobilis subsp. extranuclearis", hosted in the cytoplasm of *Paramecium nephridiatum* and *Euplotes aediculatus*, and "*Ca.* Trichorickettsia mobilis subsp. hyperinfectiva", hosted in the cytoplasm of *P. calkinsi*. According to [2], "*Ca.* Trichorickettsia mobilis subsp. extranuclearis" emerges as the sister-clade to "*Ca.* Trichorickettsia mobilis subsp. mobilis", while "*Ca.* Trichorickettsia mobilis subsp. hyperinfectiva" is the earliest divergent. In the original definition, each subspecies was characterized by a single subcellular localization, but in the present work a strain closely related to "*Ca.* Trichorickettsia mobilis subsp. hyperinfectiva" was instead found in the macronucleus of *P. multimicronucleatum*. Thus, an alternative interpretation seems to be that the location depends on the host species, as observed also in other ciliate symbionts [3]. It is now evident that there are also differences among and within the three subspecies concerning the presence/absence of: i) cytoplasmic electron-dense particles, ii) flagella, and iii) motility. "*Ca.* Trichorickettsia mobilis subsp. mobilis" and "*Ca.* Trichorickettsia mobilis subsp. extranuclearis" from *P. nephridiatum* share the presence of regularly arranged electron-dense particles in their cytoplasm while this character has not been observed in "*Ca.* Trichorickettsia mobilis subsp. hyperinfectiva". The presence of flagella has been observed in both "*Ca.* Trichorickettsia mobilis subsp. mobilis" and "*Ca.* Trichorickettsia mobilis subsp. hyperinfectiva" irrespective of endosymbiont localization, while they lack in "*Ca.* Trichorickettsia mobilis subsp. extranuclearis". Motility seems to be a shared feature between the two flagellated subspecies, although with some differences, as all macronuclear representatives of both subspecies were clearly motile inside host cells, while specimens of the "*Ca.* Trichorickettsia mobilis subsp. hyperinfectiva" in the cytoplasm of *P. calkinsi* appeared densely packed and nearly motionless, but they showed some motility after release from squashed ciliate cells [2].

In terms of morphological variability, "*Ca.* Trichorickettsia mobilis subsp. hyperinfectiva" endosymbionts in *P. calkinsi* differ in cell shape and dimensions with respect to the endosymbionts of *P. multimicronucleatum*, the former being longer and slender than the latter

(sizes: 2.5-3.0 x 0.25-0.35 µm vs ~ 1.2--2.1 x 0.5--0.6 µm). Additional differences occur among them, such as the apparently lower number of flagella of the endosymbionts of *P. multimicronucleatum* macronucleus. Unlike flagella of endosymbionts in *P. calkinsi* cells [2], in our study flagella were especially detected after negative staining (Fig. 3), being less evident, but still appreciable, in resin-embedded ciliates only (Figs. 2A, 2C, 2D). However, these flagella are quite thin and short, thus, they might also have been not well preserved and/or overlooked in some TEM preparations (see for instance [4]). Nevertheless, during TEM analysis of antibiotic-treated planarians subjected to transfer experiment, they were detected around transferred bacteria in planarian intestine cells (Figs. 5B, 5F).

To sum up, some morphological plasticity seems to arise as a characteristic of "*Ca.* Trichorickettsia mobilis" supporting suggestions of previous studies [1], i.e. in this endosymbiont species flagella are not always exhibited and are likely induced possibly driven by the host species. Additionally, flagella are subjected to variability in density and distribution on bacterial cell, with differences occurring among subspecies and even among specimens of closely related strains with different endosymbiont localizations inside the host cells.

Flagella are involved in bacterial motility, and the movement of many endosymbionts was appreciable in our study both during *in vivo* observation of intact *P. multimicronucleatum* cells and after ciliate cell squashing and bacteria releasing (L. Modeo, 2017, personal observations). In this context, one could also hypothesize that there is some correlation between the apparent morphological variability in flagellar structure of "*Ca*. Trichorickettsia mobilis" and putative functional differences in motility. For example, macronuclear endosymbionts, such as the one of *P. multimicronucleatum* US_Bl 16I1, could need less dense and shorter flagella to move inside a more delimited and more controlled place in comparison with cytoplasm, where the distances are huger and cyclosis are present. However, in the absence of experimental tests, any conclusion on this point seems premature, especially if considering that "*Ca.* Trichorickettsia mobilis subsp. mobilis" shows very dense, long flagella and a completely different movement inside the macronucleus of *P. multimicronucleatum* [1]. Interestingly, multiple flagellar morphologies were observed also among other *Rickettsiales* [3, 5].

Additionally, both the previous description of "*Ca.* Trichorickettsia mobilis" by [2] and the present study report the occurrence of a tail-like structure formed by flagella arising from cell end. The tail-like structure was typically twisted and resulted necessary for bacterial movement in

cytoplasm of *P. calkinsi*, given that bacteria lacking a "tail" were immobile according to [2]. Unfortunately, we were not able to infer whether the putative tail of flagella visible in some TEM sections (e.g. in Fig. 2D) is likewise necessary for endosymbiont movement within ciliate macronucleus of *P. multimicronucleatum*.

References

1. Vannini C, Boscaro V, Ferrantini F, Benken K, Mironov T, Schweikert M, et al. Flagellar movement in two bacteria of the family *Rickettsiaceae*: a re-evaluation of motility in the evolutionary perspective. PloS ONE. 2014. doi: 10.1371/journal.pone.0087718.
2. Sabaneyeva E, Castelli M, Szokoli F, Benken K, Lebedeva N, Salvetti A, et al. Host and symbiont intraspecific variability: the case of *Paramecium calkinsi* and "*Candidatus* Trichorickettsia mobilis". Europ J Protistol. 2018; 62: 79-94. doi: 10.1016/j.ejop.2017.12.002.
3. Lanzoni O, Sabaneyeva E, Modeo L, Castelli M, Lebedeva N, Verni F, et al. "*Candidatus* Megaira" a widespread bacterial endosymbiont of ciliates and other protists: description of a new species, environmental distribution, and phylogenetic revision. Sci Rep. 2019; 9: 1179. doi: 10.1038/s41598-018-37629-w.
4. Mariconti M, Epis S, Sacchi L, Biggiogera M, Sassera D, Genchi M, et al. A study on the presence of flagella in the order *Rickettsiales*: The case of "*Candidatus* Midichloria mitochondrii". Microbiology. 2012; 158: 1677-83. doi: 10.1099/mic.0.057174-0.
5. Boscaro V, Schrallhammer M, Benken KA, Krenek S, Szokoli F, Berendonk TU, et al. Rediscovering the genus *Lyticum*, multiflagellated symbionts of the order *Rickettsiales*. Sci Rep. 2013; 3: 3305. doi: 10.1038/srep03305.
